# Triboelectric microplasma powered by mechanical stimuli

**DOI:** 10.1038/s41467-018-06198-x

**Published:** 2018-09-13

**Authors:** Jia Cheng, Wenbo Ding, Yunlong Zi, Yijia Lu, Linhong Ji, Fan Liu, Changsheng Wu, Zhong Lin Wang

**Affiliations:** 1 School of Materials Science and Engineering, Georgia Institute of Technology, Atlanta, GA 30332-0245 USA; 2 State Key Laboratory of Tribology, Department of Mechanical Engineering, Tsinghua University, Beijing, 100084 China; 3 Department of Mechanical and Automation Engineering, The Chinese University of Hong Kong, Shatin, N.T., Hong Kong, SAR China; 4 Beijing Institute of Nanoenergy and Nanosystems, Chinese Academy of Sciences, Beijing, 100083 China; 5 School of Nanoscience and Technology, University of Chinese Academy of Sciences, Beijing, 100049 China

## Abstract

Triboelectric nanogenerators (TENGs) naturally have the capability of high voltage output to breakdown gas easily. Here we present a concept of triboelectric microplasma by integrating TENGs with the plasma source so that atmospheric-pressure plasma can be powered only by mechanical stimuli. Four classical atmospheric-pressure microplasma sources are successfully demonstrated, including dielectric barrier discharge (DBD), atmospheric-pressure non-equilibrium plasma jets (APNP-J), corona discharge, and microspark discharge. For these types of microplasma, analysis of electric characteristics, optical emission spectra, COMSOL simulation and equivalent circuit model are carried out to explain transient process of different discharge. The triboelectric microplasma has been applied to patterned luminescence and surface treatment successfully as a first-step evaluation as well as to prove the system feasibility. This work offers a promising, facile, portable and safe supplement to traditional plasma sources, and will enrich the diversity of plasma applications based on the reach of existing technologies.

## Abstract (web summary)

Gas discharge plasma sources are bulky and of limited use in remote areas with no external power supply. Here the authors create triboelectric plasma by triggering TENGs with mechanical stimuli and discuss its application as a portable plasma source.

## Introduction

Plasma, the forth state of matters, plays an important role in many fields including but not limited to nuclear fusion^1,2^, laser^3,4^, semiconductor^5–7^, display^8,9^, biomedicine^10–13^, nanotechnology^14–16^, surface treatment^17–19^, and aerospace^20^. For different applications, a variety of plasma sources have been created. But in most cases, they need to be generated and sustained through external electrical power sources, whose mobility is restricted by either the connection to power grid or batteries with limited capacity^21,22^. This greatly hinders the applications of plasma in scenarios where electrical power is scarce, such as wound treatment in the wild or emergency^13,21,23,24^, and dust removal or thrust corrections on Mars^25,26^. Existing solutions include piezoelectric direct discharge (PDD) plasma, solar-powered plasma and triboplasma^23,27–31^. PDD plasma eliminates the need of traditional high voltage transformer but still requires a power supply as energy input^27,28^. The solar-powered plasma needs a transformer and an energy storage device for dark environment^23^. Triboplasma, induced near contact point where the diamond slides on sapphire surface under pressure, is limited to in situ applications^29–31^. Therefore, the investigation on portable and self-powered plasma generation using ambient mechanical energies is impending and worthwhile, even though it is both scientifically and technically challenging.

Recently, triboelectric nanogenerators (TENGs), originating from the displacement current in the Maxwell’s equations where the contact electrification is taken into account^32^, has been developed and successfully demonstrated in numerous self-powered applications^33–37^. A born unique character of the TENG is high voltage, which has been utilized recently to quantitatively generate the input ions in mass spectrometry^38^ and fabricating of electrospun nanofibers^39^. Actually, discharges are very commonly observed in the TENGs operation when a high enough electric potential difference (EPD, ~kV) is built up^40,41^, which indicates TENG can be probably utilized to controllably induce continuous electrostatic discharge for plasma generation.

Therefore, we, present a concept of triboelectric plasma by combining plasma source with TENGs and realize atmospheric-pressure plasma with mechanical stimuli. A high enough voltage induced by triboelectrification is utilized to break down gas for plasma generation at remoted location/distance. This approach might open up possibilities for direct application of TENGs in high-voltage fields, particularly in self-powered plasma. In consideration of potential applications and electric characteristics of TENGs, atmospheric-pressure microplasma sources are selected in our study.

In the following, four classical types of TENG-driven microplasma sources at atmospheric pressure, including dielectric barrier discharge (DBD), atmospheric-pressure non-equilibrium plasma jets (APNP-J), corona discharge and microspark discharge are demonstrated. The transient electric characteristics and optical emission spectra of typical triboelectric plasma are simultaneously analyzed and compared in details, both experimentally and theoretically. The influence of design parameters of the microplasma devices, including the electrodes gap, the diameter of wire electrode and the flow rate, on the discharge performance are investigated, which can provide guidance for optimization. The applications of triboelectric plasma in patterned luminescence and plasma surface treatment are demonstrated successfully as a first-step evaluation as well as to prove the system feasibility. We believe this work not only extends the applications of TENG to self-powered plasma, but also enriches the diversity of plasma applications by providing a facile and portable supplement to traditional plasma sources.

## Results

### Design and characterization of triboelectric microplasma

**Fig. 1:**
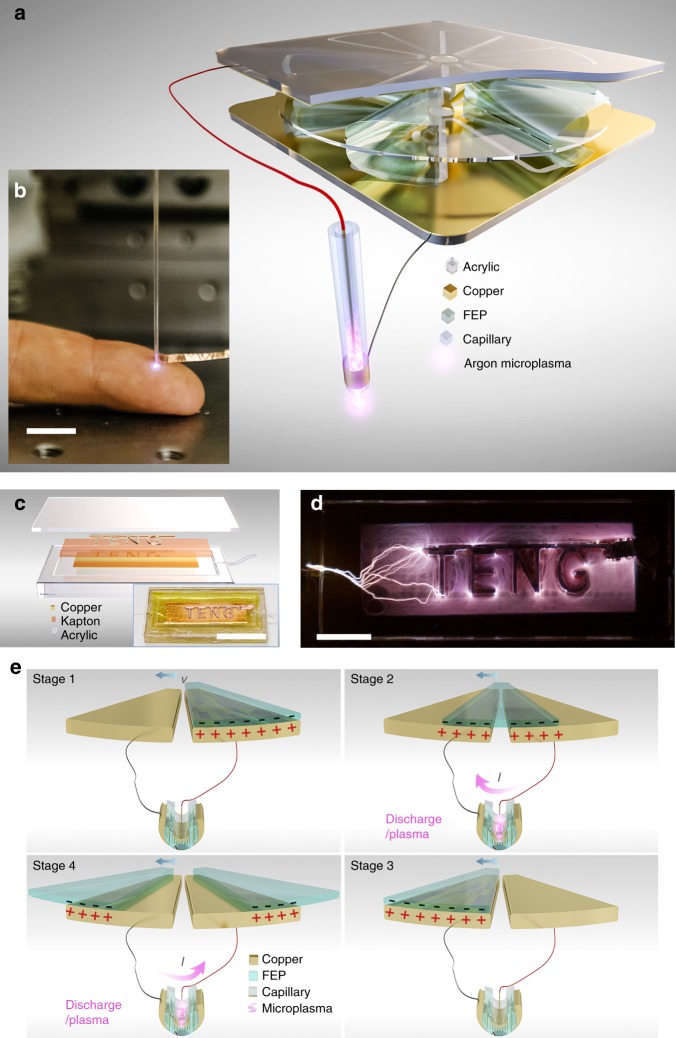
Schematic and experiment photos of triboelectric plasma. **a** Schemes of an atmospheric-pressure non-equilibrium plasma jets (APNP-J) directly driven by two serial freestanding rotary triboelectric nanogenerators (FR-TENGs). To enhance the output voltage, two FR-TENGs are mounted face to face with the same phase and the electrodes are connected in series. Each FR-TENG is composed of not only a stator coated by copper foil which is evenly divided into 12 sector as two electrodes, but also a rotator fixed of 6 fluorinated ethylene propylene (FEP) films with the other edge free on each side. The rotator has to rotate in clockwise at this point of view. The diameters of rotator and capillary are Ø295 mm and Ø0.88 mm, respectively. **b** As a low-temperature plasma, APNP-J can contact skin of human finger for biomedicine applications (scale bar, 10 mm). **c** Schematic of a patterned electrode dielectric barrier discharge (DBD) plasma source. The inset shows a photo of device (scale bar, 30 mm). **d** A luminescence photograph of patterned electrode directly driven by TENG (scale bar, 10 mm). 10 s exposure, no Photoshop (NIKON D700 @ 70 mm, ISO 1000, f/3.5). **e** Schematics of four stages in one full electric cycle at the two sector-electrodes connected with a DBD device. Blue arrow shows the velocity direction of the FEP film. There are theoretically two main discharge processes in one full cycle, in the opposite current directions

According to Paschen’s curve, it usually requires more than 1000 V to drive a typical argon discharge in atmosphere when the gap of discharge electrodes is within the magnitude of submillimeter. Meanwhile, TENGs, as an emerging technique, has been proved to be a simple, safe and effective high-voltage power source which can easily generate voltages of thousands of volts via the triboelectrification and electrostatic induction of two different materials^32^. Hence, we propose to integrate the plasma source with TENG to realize the atmospheric-pressure plasma generation via mechanical stimuli. In the practical application, to achieve relatively high voltage frequency taking durability into account, here we developed a freestanding rotary (FR) TENG device (Fig. 1a)^42–44^. The structure of the FR-TENG was optimized by considering both the best triboelectrification effect and the least wear and tear of polymer films during long-term friction. Two FR-TENGs are connected in series to enhance the output voltage, with the phase being synchronized. The fabrication process of FR-TENGs is described in details in the Method section. The microplasma source consists of a glass capillary where argon flows through in atmosphere, a metal wire electrode inside of the capillary and a copper foil electrode outside. The triboelectric microplasma generated by our setup can be easily observed by naked eyes as it is targeted at a human finger, simulating the scenario of plasma treatment (Fig. 1b). In Fig. 1c, d, a luminescence photograph of patterned electrode directly driven by TENG, where both arc discharge and filamentary discharge occurred, was demonstrated. The device photo is shown in the inset of Fig. 1c and the luminescence process is recorded in Supplementary Movie 1.

The microplasma source is directly connected to the two electrical terminals of the TENG. The working mechanism of the triboelectric microplasma is illustrated in Fig. 1e. First, the Fluorinated Ethylene Propylene (FEP) film and the right-hand side TENG electrode are in contact due to electrostatic attraction, with net negative charges on the FEP surface and positive ones on the electrode. When the FEP film slides towards the left-hand electrode, the EPD between the two electrodes, that is, the voltage applied to the microplasma device, increases^44,45^. When the electric field inside of the capillary exceeds the threshold of gas breakdown, the discharge takes place immediately with current flowing from right-hand side towards left-hand side. With the FEP film sliding forward, an opposite EPD will be established and result in another discharge with an opposite current direction. Therefore, each operation cycle should have two opposite-direction main discharges along with several small discharges.

**Fig. 2:**
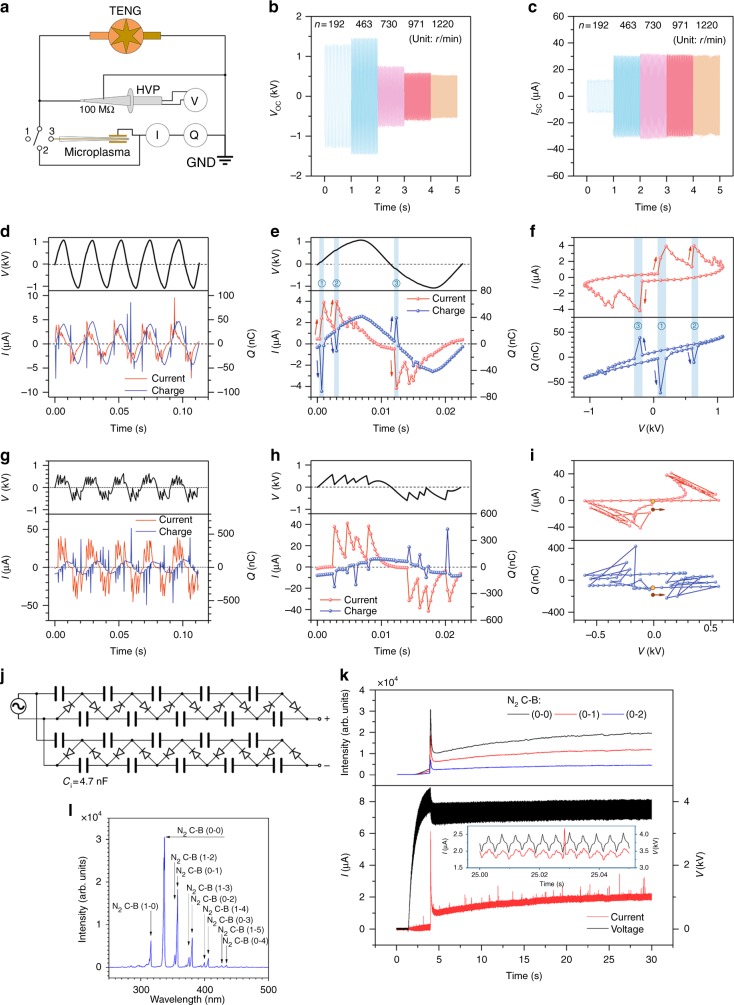
Electric characteristics of FR-TENG and discharge, and optical emission spectra of N_2_ corona discharge. **a** Circuit schematic measuring electric characteristics of triboelectric nanogenerator (TENG) and microplasma. The voltage, current and charge waveforms are measured using a high-voltage probe (HVP, Tektronix P6015A) and two electrometers (Keithley 6514, one for current, and the other for charge in different ranges), respectively. GND, electrical ground. Switching position 1, 2, and 3 means measurement of open circuit voltage, short circuit current and charge, and simultaneously electric characteristics with microplasma (as load), respectively. **b**, **c** Open circuit voltage and short circuit current of TENG with various rotational speeds. **d–f** Electric characteristic of dielectric barrier discharge (DBD) capillary plasma. **d** In five electric cycles voltage, current, and charge waveform. **e** In one electric cycle voltage, current, and charge waveform. There are three obvious discharges in the cycle. Arrows indicate the sequence of characteristics evolving. The number 1–3 and the blue gray bars correspond to three sequential discharges in (**e**). **f** Corresponding to **e**, Lissajous pattern of current–voltage and charge–voltage describing the circulation of characteristics in one full electric cycle. **g**–**i** Electric characteristics of microspark discharge, which are similar to **d**–**f**, respectively. However, voltage fluctuation in microspark discharge is more significant than DBD. In microspark discharge, the amplitudes of current and charge transferring through the electrodes gap are several orders higher than those in DBD. **j** Five-stage voltage multiplier circuit for converting AC to DC with high voltage, which is used in N_2_ corona discharge. **k**, **l** Optical emission spectra of N_2_ corona discharge. **k** Intensity of three spectral lines, voltage and current simultaneously change with time. The inset shows details of *I*/*V* around the time of *t* = 25.00 s. **l** Emission spectrum of N_2_ 2nd positive system in UV–vis region at the time of *t* *=* 4.0 s (Ocean Optics Maya2000 Pro, 200–650 nm, integration time is 100 ms.)

**Fig. 3:**
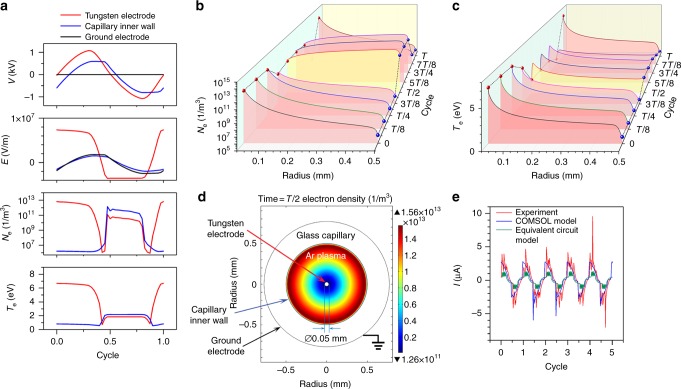
Simulation of dielectric barrier discharge (DBD) capillary plasma. **a** The transient voltage, electric field, electron density and electron temperature waveform with time at the points of tungsten electrode, capillary inner wall and ground electrode in one cycle, respectively. **b**, **c** Electron density and electron temperature profiles along the radius inside of capillary from tungsten to capillary inner wall at various typical time in one cycle. The value at the time of *T*/2 is highlighted by light yellow. **d** Time = *T*/2 (half of one cycle), electron density distribution in the cross section of the glass capillary with inner diameter of Ø1 mm and outer diameter of Ø1.54 mm. Diameter of tungsten wire electrode is Ø0.05 mm. Copper foil outside of capillary is grounded. The frequency *f* is 44 Hz. **e** Current waveforms of experiment, COMSOL model, and equivalent circuit model

Before investigating microplasma driven by FR-TENGs, the basic electric characteristics of the TENG, such as open-circuit voltage (*V*_OC_), short-circuit current (*I*_SC_) and charge (*Q*_SC_), are tested. According to the previous work, both *V*_OC_ and *Q*_SC_ should keep constant, while I_SC_ should increase with the rotational speed going up, but there is no TENG with an output voltage of above 1 kV at such a high rotational speed^43,46^. To investigate the relationship between the TENG and the microplasma, we have tested the basic performance of the TENG and the microplasma using the electrical circuit shown as Fig. 2a. Different from the previous experience, the *V*_OC_ has its maximum with a rotational speed from 192 to 1220 rounds per minute (rpm) (Fig. 2b); and the *I*_SC_ reaches the limited constant when the rotational speed is over 463 rpm (Fig. 2c). Here, the FEP film fixed with only one edge on the rotator is sweeping above the copper foil surface with small electrostatic attraction force, and will be floating up when the rotational speed rising. Thus, the efficient contact area between the FEP film and the copper foil becomes smaller than before, so that the transferred charges and the open-circuit voltage are less accordingly. However, as transferred charges per second, the current remains nearly constant at high rotational speed as shown in Fig. 2c. The *V*_OC_ below 1 kV is difficult to excite the microplasma, and hence in the discharge experiments we set the rotational speed of around 463 rpm to achieve the high enough output voltage (Fig. 2b). More details of TENG performance are shown in Supplementary Fig. 1 of Supplementary Note 1. By switching to position 3 in Fig. 2a, we can simultaneously measure the transient voltage, current and charge waveform of microplasma driven by the FR-TENGs. Figure 2d–f shows the relationship among the *V*, *I* and *Q* of a DBD capillary plasma, and the photo of device is shown in the inset of Supplementary Fig. 8d. The outer diameter of the glass capillary is only 1.54 mm, and the diameter of the tungsten wire as the internal electrode is only 0.05 mm. The external electrode around the capillary is made of copper foil with the width of 10 mm. To easily initiate the discharge, argon which has a relatively low breakdown voltage flows through the capillary at atmospheric pressure. In Fig. 2d, five cycles are shown. Typically, in these five cycles the pulsed current is up to about 10 μA, and the pulsed charge is up to about 100 nC. In Fig. 2e, the voltage waveform looks like the triangular wave as mentioned in previous work^44^, which can be simply approximated by Fourier Series with coefficient derived from the measurements (Supplementary Equation 7 in Supplementary Note 4). As shown in Fig. 2e, in one full electric cycle, there are three obvious pulsed peaks in the current and charge waveform simultaneously, which are defined as discharges. The pulsed peaks indicate that argon was broken down by the local high electric field, which result in lots of charges are transferred immediately through the circuit. The sampling frequency (*f*_*s*_ = 3 kHz) is high enough to catch the details of the discharge in this case. At the moment of the first discharge (marked as "1" in Fig. 2e), the current increased from 0.46 μA to 3.89 μA in 1/1500 s, and the charge increased from −2.78 nC to −71.32 nC within 1/3000 s, and then decreased back to −2.78 nC within another 1/3000 s. The negative sign (−) indicates that the charge flowing direction through the electrometer remains the same. There must be about 68.54 nC charges transferred from one electrode to the other in the 2/3000 s, which means that argon inside of capillary was ionized suddenly to produce microplasma and an instance discharge charge channel was established at this moment. After 5/3000 s, the second discharge occurred. Once the discharge conditions deteriorate, for example, the electric field is lower than threshold, or there is not enough “seed” charges in the zone and so on, argon molecular will be hardly excited due to lack of sufficient charged particles and energy. As a result, argon plasma will disappear quite soon. Because the frequency of the voltage is only around 44 Hz, which is determined by the rotational speed of FR-TENG, the electric field changes too slow to excite a new discharge before the microplasma is off. In addition, the quantity of transferred charges is pretty small as well. These are the reasons why in this case the microplasma is not able to sustain in a high ionization level, so that it looks not as bright as those driven by the commercial power supply. It takes 26/3000 s from the 2nd to the 3rd discharge, which is too long to sustain microplasma in good conditions of high density. In Fig. 2d, e the current is ahead of voltage, which means the load of the electrical circuit is capacitive. The Lissajous pattern of *I*–*V* and *Q*–*V* curves^47^ (Fig. 2f) helps us clarify the simultaneous relationship among the *V*, *I*, and *Q* in one cycle. Three discharges are highlighted. Corresponding to Fig. 2d–f, we conducted a DBD microplasma simulation via COMSOL and Simulink (MATLAB®) with the same experimental conditions, as shown in Fig. 3.

**Fig. 4:**
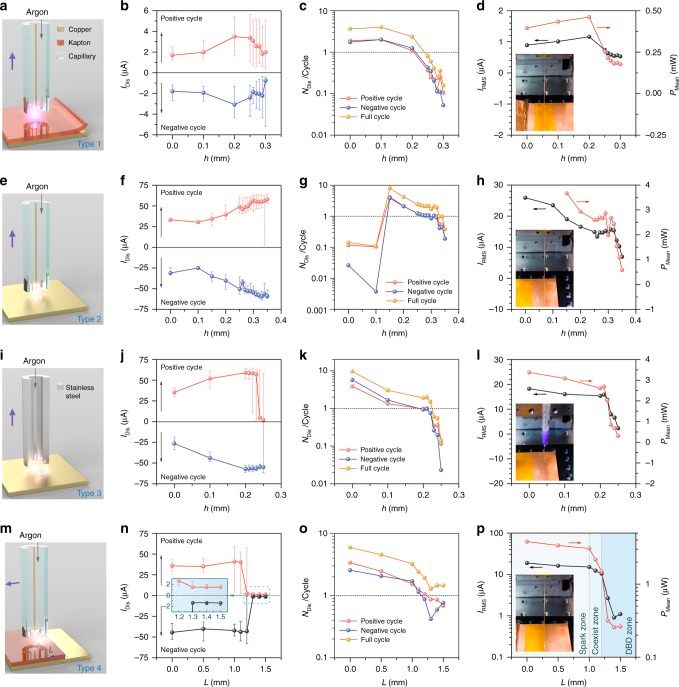
Discharge characteristics change with distance between the electrodes in different discharges. **a**–**d** In dielectric barrier discharge (DBD) plasma (type 1), discharge current, time averaged number of discharge per cycle, effective value of current and mean power change (same characteristics as follows) when lifting the capillary with the copper wire electrode from the Kapton surface. In **b**, the symbol of ball means the median value and error bar indicates the range from 10% to 90% of the whole sampling data (similar as follows). **e**–**h** In microspark discharge (type 2), there is no dielectric film between the two electrodes. At the beginning of lifting the copper wire, there is a continuous discharge path between the two electrodes which results in no pulsed discharge current detected (**g**) but high effective value of current just like short circuit current (**h**). **i**–**l** Another type of microspark discharge (type 3) with a stainless-steel capillary tube. **m**–**p** Holding the height of 0.20 mm, capillary moving horizontally from the border of copper and Kapton towards left which leads to the obvious changes of discharge mode and status from microspark to DBD plasma. **p** The current and power are both much higher in spark zone than those in DBD zone, and they decline sharply in the coexist zone

Figure 2g–i show the relationship among the *V*, *I* and *Q* of a microspark discharge (Fig. 4e, h). The outer diameter of the capillary is 0.88 mm, inside of which the diameter of the copper wire electrode is 0.08 mm. The distance between the end of capillary and the copper foil is 0.15 mm. The rotational speed of FR-TENG is the same as that in Fig. 2d–f. However, it is different from the Fig. 2d that the voltage waveform in Fig. 2g fluctuates sharply with time, as well as current and charge. Meanwhile, there are more discharges per cycle than DBD microplasma driven by the FR-TENG. Compared with Fig. 2d, in Fig. 2g the amplitude of the voltage is only 0.57 kV, and the pulsed peaks of the current and the charge amount are one order of magnitude higher than those of the DBD discharge, up to 40 μA and 500 nC, respectively. This difference might be explained as that quite more charges could be more easily transferred between the two electrodes due to absence of the dielectric layer. Transmission of so many charges has a significant influence on the output voltage of FR-TENG. This is very different from the conditions of traditional microplasma discharges using the commercial power supplies. It should be noted here that when the amount of transferring charges is too enormous, TENG is not able to supply nearly infinite charges to the microplasma source as the commercial power does.

To investigate corona discharge characteristics, a voltage multiplier circuit is employed with a 24-sector FR-TENG for converting AC to DC with high enough voltage. In theory, the DC output voltage should be promoted by five times of the AC peak-to-peak input voltage by use of the circuit shown in Fig. 2j. However, the actual output voltage is usually much lower than predicted due to the current leakage of electronic modules in the circuits. The experimental setup is similar to Fig. 2a, except that the Pintech HVP-40 was used for the voltage measurement because of its higher impedance (~1GΩ), which is good for reducing the branch current. 200 sccm nitrogen was fed through a capillary with a Ø0.04 mm copper wire, which was the same as Fig. 1a, b. An obvious corona discharge could be observed by naked eyes even under the normal ambient light. In Fig. 2l, serval characteristic spectral lines are shown in UV–vis region, which means a typical N_2_ corona discharge with 2nd positive system (C^3^Π_u_ → B^3^Π_g_) was happening.

In another experiment, the absolute intensity measurement of the UV irradiation was made for quantitative evaluation of UV intensity, resulting in the magnitude of around 0.001 μW/cm^−2^/nm (shown in Supplementary Fig. 2 of Supplementary Note 2). Though the absolute intensity of UV is very low, when the fiber is put very close to the end of the capillary, the relative intensity in UV region is high enough to be detected even in the daylight. Benefiting from the good signal-to-noise ratio (SNR), this type of corona discharge would be a promising UV (or other wavelength) light source for the applications of species detection^48^, elemental analysis^49^, micro total analysis system (μ-TAS)^49^ and gas chromatography^50^ in the future. The intensity of emission spectra changes with the plasma status, as well as the voltage and the current, as shown in Fig. 2k. The voltage multiplier circuit had been charging till at the time of *t* = 4.0 s, and at that time a strong discharge occurred resulting in a spike pulse of current and spectra, and a break-down of voltage simultaneously. The N_2_ corona discharge spectra are recorded at different time shown in Supplementary Fig. 3 of Supplementary Note 2, which is useful for the investigation of the discharge process along with the electric characteristics.

Furthermore, from the experimental observation, nitrogen is hardly ionized comparing with argon and helium, because its breakdown voltage is higher in this case. It is a difficulty and a challenge to make the triboelectric microplasma as a portable plasma source for more application without argon or helium in air circumstances. To estimate the difficulty of air discharge, we have measured the current of air discharges in the same experimental conditions except for the methods in use of TENG, which is with or without the voltage multiplier circuit, respectively. The current waveforms are presented in Supplementary Fig. 7a,b.

In addition, the efficiency from mechanical energy to electric energy is estimated in the magnitude of 0.1% in Supplementary Note 3, as shown in Supplementary Fig. 4, and Supplementary Table 1.

### Microplasma simulation

Corresponding to Fig. 2d–f, we conducted DBD argon microplasma simulations via COMSOL (Fig. 3) and Simulink (Fig. 3e), respectively, with the same experimental conditions as shown in Supplementary Fig. 8d. In the COMSOL simulation, a plasma model with seven reaction formulas are adopted (simulation setup in details listed in Supplementary Note 4 of the supplementary information). Along the radius of the capillary, there are three important positions monitored with time, which are the tungsten wire surface as the inner electrode, the capillary inner wall and the copper foil as the ground electrode, respectively. Variations of voltage, electric field, electron density and electron temperature at the three positions with time in one full cycle are shown in Fig. 3a. At the beginning, the voltage of tungsten electrode is in the positive cycle and higher than the voltage of capillary inner wall, resulting in more electron gathering near the tungsten electrode. And then the EPD between the tungsten electrode and capillary inner wall inverts in the opposite direction. Electron near the tungsten electrode gains more energy to accelerate due to the electric field, and then excited argon plasma inside of capillary. The electron density and temperature at tungsten electrode and capillary inner wall both change simultaneously with time in the same order, which means that the zone inside of capillary is full of plasma. However, the argon plasma cannot be sustained for a long time because of low energy and ionization, and there is one maximum peak density at the time of *T*/2 (half cycle) as shown in Fig. 3b. The similar phenomenon of several obvious discharges has been observed in the experiment as shown in Fig. 2d, e. In addition, electron temperature profiles along the radius at various typical moments are shown in Fig. 3c. The max value (6.69 eV) exists in the sheath region near the tungsten wire as the anode where there are few electrons due to weakly ionized microplasma, which is far from equilibrium^51^. The electron density in this DBD microplasma model in the order of 10^13^ /m^3^ is quite lower than those published before (10^16^–10^17^/m^3^)^52^. Meanwhile the light emitted by the argon microplasma in the experiment is too dull to be almost visually observed, which is a proof of the low electron density as well. At the time of *T*/2, the electron number density reaches the maximum of 1.56 × 10^13^/m^3^, of which the distribution across the radius of the capillary is shown in Fig. 3d and highlighted by the color of light yellow in Fig. 3b as well. The *N*_e_ changes in a full cycle is shown in Supplementary Movie 2. The voltage waveform of the tungsten electrode as the input of COMSOL model is the same as the equation of triangle waveform (44 Hz) in the type of Fourier Series from the experiment (shown in Supplementary Equation 7 in Supplementary Note 4). The experimental and approximate simulation voltage waveforms are shown in Supplementary Fig. 5, which are consistent with each other. In Fig. 3e, the current of the COMSOL model is in accordance with the experimental results.

Meanwhile, there is another current waveform in the same figure, which is derived from the equivalent circuit model calculated in Simulink based on the model in the reference^53^. In this model, the microplasma is considered as a variable capacitor when discharge occurs. The capacitance of the microplasma device has been measured as of around 2.35 pF by means of LCR meter (NF ZM2371, with ZM2325AM), although the calculated capacitance is only 0.188 pF in theory according to the reference^53^. This might be the reason that wire electrode is actually put very close to the inner wall of capillary, which is not axial symmetric configuration. However, the simulation results agree with the experiments in phase except for the half of magnitude (Fig. 3e). Different from the equivalent circuit of the reference^53^, a resistance of 1 GΩ is connected to the main circuit in parallel, which means the inner impedance of the high voltage probe. More details of the equivalent circuit model are presented in Supplementary Note 4, and the parameters of capacitance and resistance are listed in Supplementary Table 2.

### Analysis of discharge parameters

In practice, there are many vital methodological and configuration parameters that can affect the discharge performances significantly. To investigate influence of various parameters on microplasma characteristics and help optimize our device, experiments were carried out by controlling the gap between two electrodes, the diameter of wire electrode, and the gas flow rate, which are the three vital parameters for plasma generation. The system performance with different discharge modes and gaps between two electrodes was evaluated and demonstrated in Fig. 4, and the performance with various diameters of the wire electrode and argon gas flow rates can be found in Supplementary Figs. 8, 9, respectively.

In Fig. 4a, a capillary (Ø0.88 mm) with a copper wire (Ø0.08 mm) was lifted from the surface of Kapton, initial position of *h* = 0. There was a spot of microplasma on the tip of the wire. The discharge currents (*I*_Dis_) in positive and negative cycles are both shown in Fig. 4b. An index, time average number of discharge per cycle (*N*_Dis_/Cycle) was introduced to evaluate the difficulty of discharge (shown in Fig. 4c). Meanwhile the *N*_Dis_/Cycle in the positive and negative half cycles were compared as well. At the beginning discharge occurred 3.64 times per full cycle in average. The *N*_Dis_/Cycle almost kept constant and then declined with the height, especially above the height of 0.25 mm, the *N*_Dis_/Cycle was <1, which means that discharge started to become difficult. There is generally only one discharge within several cycles. When we lift the capillary to the height of 0.3 mm, there is hardly discharge at all. The effective value of current (*I*_RMS_) is also introduced to compare the performance of discharge, which is the root mean square (RMS) value of instant current. The *I*_RMS_ under the conditions of type 1 is around 1 μA. The mean power (*P*_Mean_) is calculated by the time average of instant power (*P*(*t*) = *V*(*t*)×*I*(*t*)). In Fig. 4d, the range of *P*_Mean_ is from 0.15 to 0.5 mW.

When we remove the Kapton film from the type 1 in Fig. 4a, the tip of copper wire directly faces the copper foil electrode as the type 2 shown in Fig. 4e. Microsparks were occurring instead of DBD microplasma at the discharge spot. At the beginning of lifting from *h* = 0 to *h* = 0.1 mm, no obvious discharge was observed just because short circuit might be occurred. The median of *I*_Dis_ increases with the height after *h* = 0.1 mm (Fig. 4f), however the *N*_Dis_/Cycle decreases. The *N*_Dis_/Cycle is up to 8.02 which is much higher than in type 1 as shown in Fig. 4g. Then *I*_RMS_ and *P*_Mean_ (shown in Fig. 4h) are up to 19 μA and 3.67 mW respectively except for the condition of short circuit, which are both one order of magnitude higher than those in type 1. From the experiments of type 2, we can deduce that microspark transfers more charges and dissipates more energy per unit time than DBD microplasma.

The main difference between the type 2 and type 3 (Fig. 4i) is the upper electrode, where the former is copper wire inside of a capillary, and the latter is stainless steel capillary tube (as microhollow electrode) with diameter of 0.30 mm. The basic electric characteristics of type 3 are shown in Fig. 4j–l. When the tip of capillary tube passes the height of *h* = 0.23 mm, the *N*_Dis_/Cycle declines sharply to 0.61. Actually, the process of lifting the capillary tube from the surface of copper foil had been recorded by camera as shown in Supplementary Movie 3, in which we can find that the length of the microsparks had been pulled longer and longer gradually. The voltage and current of a typical discharge within five cycles are shown in Supplementary Fig. 6. The electric characteristic measurement and analysis of other types of discharges are presented in Supplementary Note 5.

To investigate the transient process from microspark converting to DBD microplasma, we designed an experiment as the type 4 shown in Fig. 4m. Holding a capillary with copper wire inside on the height of *h*_0_ = 0.20 mm, we moved horizontally the capillary from the border of copper and Kapton (*L* = 0) towards left (*L* > 0). The process had been recorded in Supplementary Movie 4. At the beginning, there are typical microspark with multi-discharge, and then the microspark is pulled as long as the distance of *L*, while the number of discharge decreases. The similar phenomenon appears in Fig. 4o as well. There is a sharp decline of the *I*_RMS_ and the *P*_Mean_ in Fig. 4p from *L* = 1.0 mm to *L* = 1.3 mm. That’s because the mode of discharge changes to DBD microplasma. From the Fig. 4n, at the distance of *L* = 1.0 mm, the amplitude of *I*_Dis_ in positive cycle starts to appear as little as typical DBD plasma in the order of several microamperes, although the major mode of discharge at this moment is still microspark (the median *I*_Dis_ is 40.9 μA). The same situation is at the distance of *L* = 1.1 mm. Whereas at *L* = 1.2 mm, the range of *I*_Dis_ in positive cycle is from 1.8 μA to 57.5 μA, and the median value is 2.57 μA which means the major discharge is becoming DBD plasma despite partial microspark. At *L* = 1.3 mm there is no more microspark. As a result, we define the zone less than *L* = 1.0 mm as the Spark Zone, more than *L* = 1.2 mm as the DBD Zone, however the zone between *L* = 1.0 and *L* = 1.2 mm is called Coexist Zone which indicates both microspark and DBD plasma appear.

In Supplementary Fig. 8 we demonstrate the electric characteristics of DBD microplasma with the diameter of wire electrode. The device is shown in the inset of Supplementary Fig. 8d. We can easily find that the diameter of wire electrode (from 0.04 to 0.15 mm) is not so much significant to influent the discharge performance, different from what we thought before. However, tungsten wire has better discharge performance than copper wires even the finer one. Electron are more easily emitted from tungsten than copper.

In Supplementary Fig. 9 the influence of the argon gas flow rate on discharge performance is investigated as well. There is not too much difference in the electric characteristics of microplasma once argon gas is fed through the capillary even though the gas flow rate is as low as 15 sccm. However, it is quite different from that with no argon gas feeding, because discharge hardly appears at all.

### Applications in luminescence and surface treatment

**Fig. 5:**
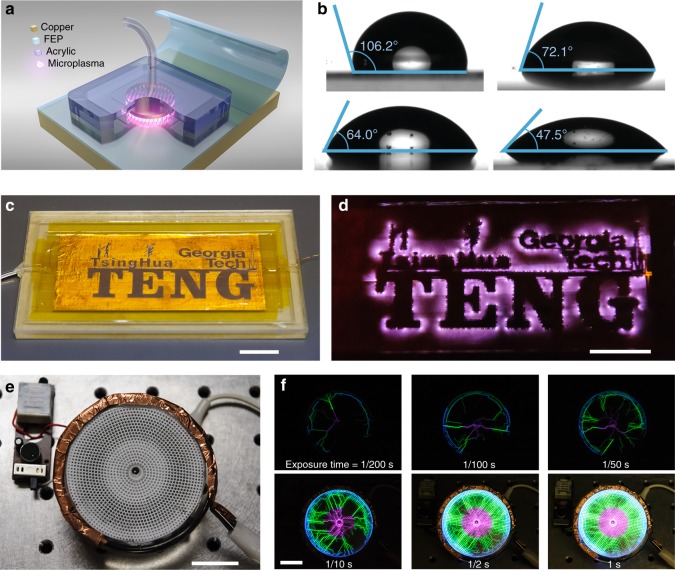
Applications of triboelectric plasma in surface treatment and luminescence. **a**, **b** Contact angle of fluorinated ethylene propylene (FEP) surface before and after microplasma treatment. **a** Schematic of surface treatment in use of triboelectric plasma. **b** Untreated surface, 1 min, 3 min, and 6 min treated by argon plasma, respectively. **c**, **d** Example of microplasma luminescence photograph showing patterns of triboelectric nanogenerator (TENG), Tsinghua, and Georgia Tech (scale bar, 20 mm). **d** No Photoshop (NIKON D700 @ 70 mm, ISO 6400, 30 s, f/2.8). **e**, **f** A plasma disk driven by freestanding rotary (FR) TENGs (scale bar, 20 mm). **f** Photos with different exposure time (NIKON D700 @ 70 mm, ISO 6400, f/7.1)

Triboelectric microplasma can be used in most application fields where traditional plasma could be used for. For example, atmospheric-pressure low temperature plasma is often used in treatment of material surface for diverse functionality. A schematic of FEP film surface treatment with microplasma to modify the hydrophobicity is shown in Fig. 5a. Contact angle is a usual measurement index of static hydrophobicity. Figure 5b shows the treatment results by triboelectric plasma in the form of the contact angle on FEP film surface. In Fig. 5b, 106.2° means the surface is typically hydrophobic. After exposure in argon triboelectric plasma for 1, 3, and 6 min, contact angle decreases to 72.1°, 64.0°, and 47.5°, respectively. The surface of FEP film obviously changes from hydrophobicity to hydrophilicity due to triboelectric plasma treatment for a while. This experimental application predicts that triboelectric microplasma also has capability of surface modification and functionality.

As shown in the above experiments, triboelectric microplasma could emit a dull light. We fabricated another patterned argon triboelectric microplasma source device as shown in Fig. 5c. Figure 5d exhibits its raw photograph with 30 s exposure in darkroom. In addition, a plasma disk can be powered by FR-TENG as well (Fig. 5e, f), and Supplementary Movie 5). As a result, luminescence and display of triboelectric microplasma could be used in various application with only mechanical stimuli, if we design and fabricate different electrodes in scale, shape and materials etc. At last, we demonstrate luminescence photos of DBD capillary plasma by means of optical microscopy. (Supplementary Fig. 10)

## Discussion

Here we demonstrated microplasma generation as excited by triboelectrification. Several different types of microplasma and discharge directly or indirectly driven by TENG were achieved successfully. This opens up possibilities for using TENG to generate diverse microplasma. For triboelectric microplasma, analysis of electric characteristics, optical emission spectra and simulation were carried out to explain transient process of different discharge. It was observed that UV radiation could be emitted in N_2_, Ar and H_2_ by triboelectric microplasma. The absolute and relative intensity of triboelectric microplasma have been measured. Some important factors of plasma sources, such as distance between electrodes, diameter of electrodes, gas flow rate and so on, were investigated for understanding their influence on microplasma and discharge performance. Triboelectric plasma provides a novel supplement to utilize plasma with no additional power supplies required. Furthermore, introducing rectifier or transformer etc. could improve characteristics of triboelectric plasma. Taking natural advantage of TENG, high voltage, low current and charge, triboelectric microplasma might has enormous potential value in applications of individual cell culture, treatment and apoptosis, species detection, elemental analysis, and ultraviolet excimer etc. In the future, triboelectric plasma science and technology could be combined with different disciplines to generate various plasma sources.

## Methods

### Fabrication of TENGs

The first FR-TENGs assembly consists of three parts, that is, two same stators as electrodes, and one rotator as shown in Fig. 1a. The stator is constructed by attaching a copper foil (40 μm) to an acrylic board (0.25 inch thick, 295 × 295 mm^2^). There is only one layer copper foil which has been evenly divided into twelve sectors. In the same layer, the twelve sectors which are connected by inner ring and outer ring respectively belong to two electrodes at intervals. The distance between two stators could be adjusted. The diameter of rotator (0.125 inch thick) is 295 mm as well. As mentioned above, FEP film (120 μm) which are fixed on both sides of rotator was cut into certain shape to cover sector with the free part. Once the direction of FEP film bending is determined, rotator is able to rotate only towards one direction. Rotator is connected to motor shaft via a flange-mount shaft collar.

The second FR-TENG is very similar as the first one, except for 24 sectors and the different friction materials (FEP film adhered on the copper of the stator, Nylon film fixed on the rotator). Only one FR-TENG is employed for the input of voltage multiplier circuit to generate N_2_ corona discharge.

### Fabrication of microplasma devices

There are four types of DBD capillary microplasma device. The first type is shown in Fig. 1a, b. A copper wire (Ø0.04 mm) is put inside of a capillary (Drummond Scientific, Ø_outer_ 0.88 mm, Ø_inner_ 0.4 mm) as an electrode. One end of capillary is fed with argon gas by a flexible tubing (Ø_outer_ 1.65 mm), and the other end is directly exposed into atmosphere. A band of copper foil (40 μm) with width of 1 mm is wrapped around the capillary 3 mm above the outlet. The second type is shown in Supplementary Fig. 8d, resulting in Fig. 2d–f. A capillary (Drummond^®^) with diameter of 1.54 mm is laid on the acrylic board (75 × 25 mm^2^) which has a trench in width of 1.9 mm. A tungsten or copper wire with different diameters is put inside of a capillary as an electrode. Two 70 mm long flexible tubing (Ø_outer_ 1.65 mm) are connected to the capillary, one end as argon inlet, and the other end for extending outlet away from atmosphere. Copper foil with width of 10 mm is wrapped around the capillary. The third type is shown in Fig. 4a, m, the dielectric of DBD plasma is not the glass of capillary but the Kapton film (60 μm) on copper electrode, which is put vertically against the outlet of capillary. The forth type looks like the second type, the difference is copper foil as outer electrode, which is wider and wrapped around the capillary only half a circle. (Supplementary Fig. 10a)

There are two similar DBD microplasma devices with patterned electrode. One is 75 × 40 mm^2^ (Fig. 1c, d), the other is 148 × 78 mm^2^ (Fig. 5c, d). They both consists of six parts, which are base, cover, basic electrode, patterned electrode, dielectric, and tubing respectively. The base is made of Acrylic with 0.25 inch thick, however the cover is 0.125 inch thick Acrylic. In Fig. 1c, a 65 × 26 mm^2^ rectangle is engraved in depth of about 2 mm to form a chamber for plasma. A copper foil tape (40 μm) is adhered to the bottom of chamber as basic electrode. Kapton tape (60 μm) is then entirely covered the whole copper electrode as dielectric. A 1 mm-thick patterned Acrylic coated by copper as the upper electrode is put on Kapton. And then a tubing as inlet of argon gas are stuck on the trench of the base. The two electrodes have to be connected by copper wires before the cover is finally sealed on the base. In Fig. 5c, except for dimension, the main difference is the patterned electrode which is cut from a 120-μm-thick FEP film coated by phsical vapor deposition (PVD) of 10 μm Cu.

There are two microspark discharge devices. In Fig. 4e, h, one is made of a capillary (Ø0.88 mm) with a copper wire (Ø0.04 mm) inside. In Fig. 4i, l, the other is a stainless-steel capillary tube (Ø_outer_ 0.30 × 13.00 mm).

Microplasma device precisely moving. In Fig. 4, microplasma sources were all mounted on the 3-D manual linear stage (Newport ULTRAlign^TM^, 462-XYZ-M), which has sensitivity of 1.0 μm.

### Circuit and measurement of electric characteristics

The voltage was measured by means of two different high-voltage probes (HVP, Tektronix P6015A, 1/1000, 100MΩ; Pintech HVP-40, 1/1000, 1GΩ), meanwhile current and charge were achieved through two programmable electrometers (Keithley 6514, one for current, and the other for charge in different range), respectively. There is a part of current flows through the HVP, because the internal impedance is not high enough to make this branch exactly as open circuit. However, the measurement of the voltage over the microplasma source device as a load is still accuracy. To avoid zero drift when the two electrometers were connected with each other to measure current and charge simultaneously, the electrical ground of electrometer should be disconnected. The measurement signals are input high-speed data acquisition system under LabView control.

## Electronic supplementary material

Supplementary Information

Description of Additional Supplementary Files

Supplementary Movie 1

Supplementary Movie 2

Supplementary Movie 3

Supplementary Movie 4

Supplementary Movie 5

## Data Availability

The data that support the plots within this paper and other findings of this study are available from the corresponding author upon reasonable request.
