# Supplementary material for: Triboelectric microplasma powered by mechanical stimuli
Source: Nat Commun. 2018 Sep 13;9:3733. doi: 10.1038/s41467-018-06198-x (PMC6137053; doi:10.1038/s41467-018-06198-x)
Supplement: Supplementary file 1 — Supplementary Information [file 41467_2018_6198_MOESM1_ESM.pdf]

Supplementary Information:

Triboelectric microplasma powered by mechanical stimuli

Cheng *et al.*

Supplementary Note 1: Triboelectric nanogenerator (TENG) performance

From Fig. 2(b, c), Supplementary Figure 1(a~d), we can obviously find that the peak-to-peak value of voltage, current and charge with the microplasma source (load) at different rotational speeds are all lower than the corresponding peak-to-peak value without load, i.e., V_{OC}, I_{SC} and Q_{SC}. Taking the rotational speed of 463 rounds per minute (rpm) as examples, we compare the difference of electric characteristics with or without load. In Fig. 2b and Supplementary Figure 1b, the V_{pp} with load is only 2.01 kV which is much lower than the V_{pp} without load of 2.87 kV. In Fig. 2c and Supplementary Figure 1c, the I_{pp} of short circuit is as high as 61.36 µA, however, the I_{pp} with load which is able to discharge in each cycle is only 10.69 µA. Because of discharge, the microplasma device which results in lots of pulsed peaks in the current waveform is very different from the common resistance or capacitance load. The I_{pp} with load is counted by those pulsed peaks. In Supplementary Figure 1d, the Q_{pp} of short circuit is up to 272 nC, and the Q_{pp} with microplasma is only 124 nC. The electric characteristics of triboelectric plasma is quite complicated and different from the previous work ever in which various resistors are usually used as load to evaluate the performance of TENGs.

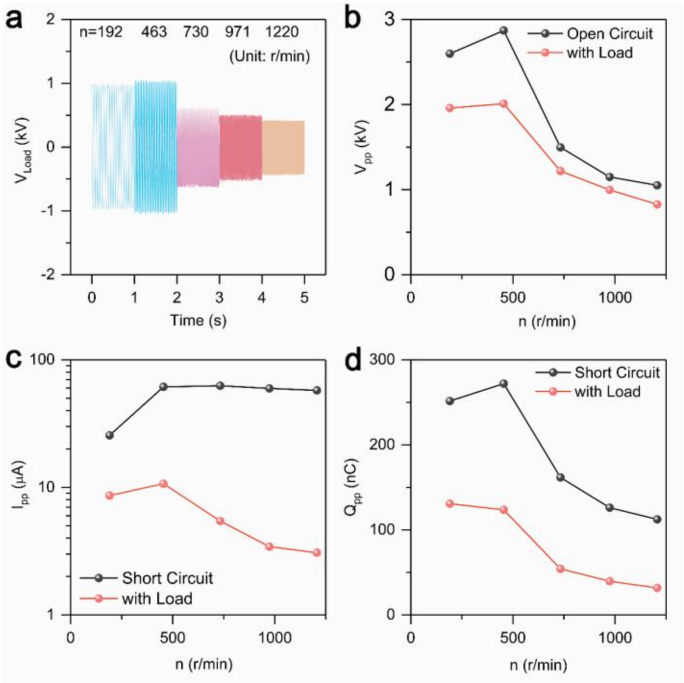

Supplementary Figure 1 | Electric characteristics of triboelectric nanogenerator with or without microplasma (load). (**a**) Voltage over load with various rotational speeds when switching position 3 in Fig. 2(a). (**b**) The peak to peak voltage (V_{pp}) with various rotational speeds under the conditions of open circuit and with load. (**c**) (**d**) The peak to peak current (I_{pp}) and charge (Q_{pp}) with various rotational speeds under the conditions of short circuit and with load, respectively.

Supplementary Note 2: Optical emission spectra of microplasma

The absolute intensity of the UV irradiation is very weak to be hardly detected, especially with the attenuation resulting from the cosine corrector. Despite the measurement difficulty of weak irradiation, we measured the absolute intensity of the emission spectra in the corona discharge in use of a calibrated spectrometer (Avantes, AvaSpec-HSC-TEC, 200-973nm). The calibration of the spectrometer was conducted by using a halogen lamp (calibrated by National Institute of Metrology, China). A cosine corrector was used to couple the fiber and the spectrometer to collect signal from 180° field of view. The cosine corrector is orthogonally mounted above the

capillary with the height of 14.90 mm. And the microplasma device, the fiber and the cosine corrector were put inside of a dark box to avoid the disturbance from ambient light. The absolute irradiation intensity of argon, nitrogen and helium corona discharge driven by TENG (via a voltage multiplier) are shown in Supplementary Figure 2. As we can see, in Supplementary Figure 2a, the absolute irradiation intensity in the UV range is very low, around 0.001 $\mu W/cm^{-2}/nm$, which are the characteristic spectral lines of nitrogen microplasma driven by corona discharge due to air breakdown near the outlet of the capillary. However, the value within the visible and NIR (Near-Infrared-Ray) range are higher, around 0.018 $\mu W/cm^{-2}/nm$, which are the characteristic spectral lines of argon microplasma. In Supplementary Figure 2(b,c), the spectra of nitrogen and helium microplasma are shown with very low absolute intensity as well. Even so, the relative intensity of UV shown in Fig. 2(k, l) and Supplementary Figure 3 is still obvious with good signal-to-noise ratio (SNR).

The triboelectric microplasma can emit different wavelengths of photons by feeding different gases. Taking nitrogen as an example, imagine that, when a little bit sample gas (or droplet) is fed into the capillary, or a target cell is close to the capillary, the relative intensity of UV radiation will be changed more or less. Therefore, the change of intensity over the characteristic spectral lines are easy to be detected due to the good SNR and sensitivity, which will be very useful for the applications of species detection[1], elemental analysis[2], micro total analysis system (μ-TAS)[2] and gas chromatography[3]. Sometimes, these applications might take place in the rural area, where a portable microplasma source triggered by mechanical stimuli may be desired. Triboelectric microplasma should be a good choice for this condition. In this manuscript, we just demonstrate a phenomenon and the feasibility of triboelectric microplasma generating, furthermore, the next step should be done is the improvement of output performance and efficiency from the engineering perspective to make it more practical.

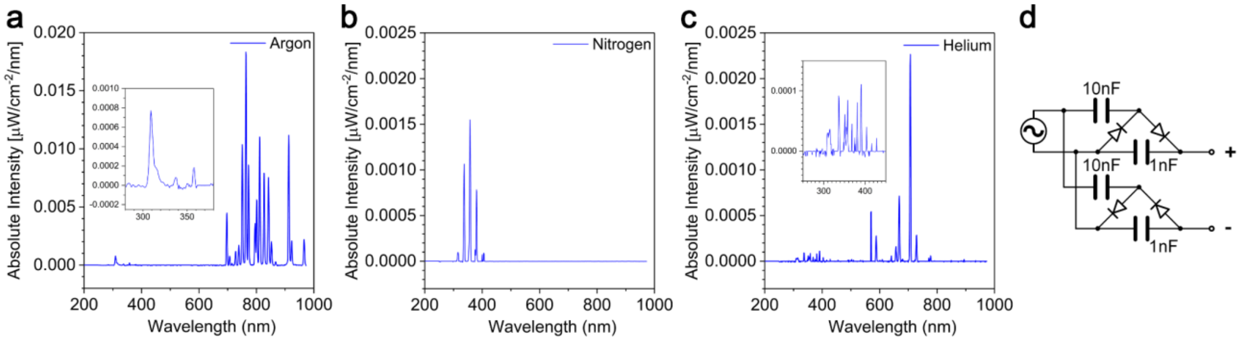

Supplementary Figure 2 | The absolute intensity of the emission spectra in the corona discharge. (**a**) Argon, (**b**) Nitrogen, (**c**) Helium, (**d**) A voltage multiplier circuit connected with triboelectric nanogenerator (TENG) is used to generate the corona discharge.

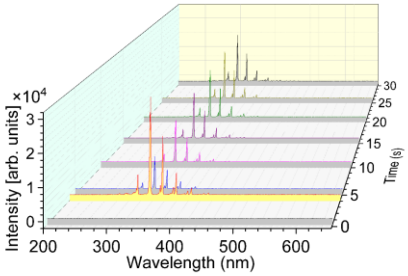

Supplementary Figure 3 | The N_2 corona discharge relative spectra at the time of *t* = 0.2, 4.0, 5.0, 10.0, …, 30.0 s. When five-stage voltage multiplier circuit is used for converting AC to DC with high voltage, N_2 corona discharge occurs. The relative spectra change with time, corresponding to Fig. 2(k, l).

Supplementary Note 3: Efficiency of converting mechanical energy to electric energy

From the experimental observation, the friction torque is too low to measure in use of general torque sensors. We fabricated a device to semi-quantitatively estimate the efficiency of converting mechanical energy to electric energy, whose schematic is illustrated in Supplementary Figure 4a. The FR-TENG is the same as the one used in N_2 corona discharge of Fig. 2k, except for the friction pair are nylon (stator) and PVC (rotator). The rotator can rotate around the shaft with the flange. There is thread whose one end is fixed on the circle of the flange, and the other end is tied to a weight of 200 g. When a weight free falling from the height of h down to the ground, TENG rotates several times resulting in fluctuation of current through a close circuit with a resistance. The height, the falling-down time, the current waveform and the resistance etc. are recorded to calculate the efficiency. One typical waveform of current is shown in Supplementary Figure 4b. The calculation process is explained as follows. As a result, the efficiency of the TENG from mechanical energy to electric energy is estimated at around 0.1%.

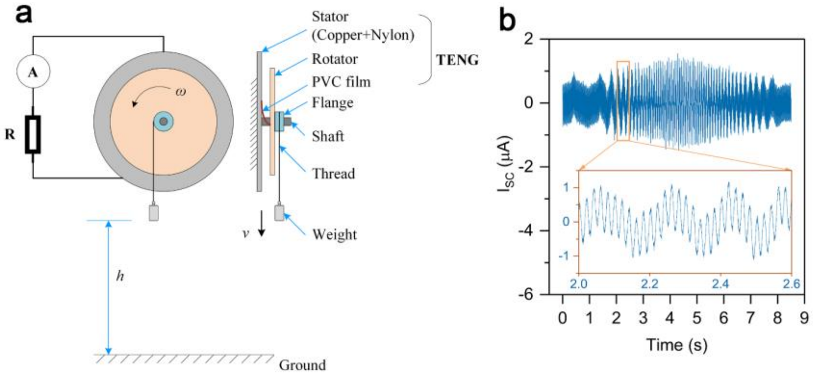

Supplementary Figure 4 | Estimate of energy converting efficiency. (**a**) Schematic of the device for measuring the efficiency of converting mechanical energy to electric energy, where A indicates current electrometer (Keithley 6514), *R* is the resistance. In (a), the left figure is the front view, and the right one is the side view. (**b**) Current waveform is measured from the beginning to the end in a typical experiment. And the inset shows the details from 2.0 s to 2.6 s.

According to the motion equations and energy conservation equation, we can list the equations as follows.

$$\frac{1}{2}at^2=h \tag{1}$$

$$v=at \tag{2}$$

$$E_{\mathrm{weight}}=mgh-\frac{1}{2}mv^2 \tag{3}$$

$$E_{\mathrm{TENG}}=I_{\mathrm{RMS}}^2Rt \tag{4}$$

$$\eta=\frac{E_{\mathrm{TENG}}}{E_{\mathrm{weight}}}\times 100\% \tag{5}$$

Where the basic parameters are, a for acceleration, v for velocity, t for time of weight falling from the beginning to the ground, h for height, m for mass of the weight, and R for resistance of the circuit, respectively; E_{weight} is the work done by the weight on the TENG; E_{TENG} refers to the electric energy generated by the TENG; I_{RMS} is the Root-Mean-Square (effective value) of current; η is the efficiency of converting energy. By substituting

Supplementary Equations 1~4 into Supplementary Equation 5, we can calculate the η with Supplementary Equation 6.

$$\eta=\frac{I_{\mathrm{RMS}}^{2}Rt}{mgh-\frac{2mh^{2}}{t^{2}}} \tag{6}$$

Three experiments were conducted, resulting in the parameters and results in Supplementary Table 1.

h/m	t/s	m/g	R/MΩ	I_{RMS}/μA	E_{TENG}/J	E_{weight}/J	η
0.585	4.6	200	510	0.52	0.0012	1.401	0.086%
0.56	3.04	200	510	0.533	0.001	1.084	0.0923%
0.56	3.21	200	510	0.536	0.001	1.0854	0.0921%

Supplementary Table 1 | Experimental parameters and efficiency of converting energy. An experiment was conducted for three times, in which a 200 g weight free falling from the height of *h* to the ground, and driving a triboelectric nanogenerator (TENG) to heat a resistance *R* of 510 MΩ with alternating current.

Supplementary Note 4: Simulation setup

The output voltage of FR-TENG is not exactly sine waveform, but like a triangular waveform with the parameters of Supplementary Equation 7 as follows, which is in the type of Fourier Series from the experiment.

$$\begin{aligned} V_{\mathrm{triangle}}(t) &= \sum_{k=0}^{\infty}(-1)^{k}a_{k}\frac{\sin(2\pi(2k+1)ft+\theta_{k})}{(2k+1)^{2}} \\ &\approx 1033.85\sin(2\pi ft+1.99\pi)-79.13\sin(6\pi ft+1.58\pi)+16.01\sin(10\pi ft+1.16\pi) \\ &\quad -1.25\sin(14\pi ft+1.30\pi)+4.27\sin(18\pi ft+1.04\pi) \end{aligned} \tag{7}$$

Where k is the number of harmonics, a_k and θ_k are the coefficients from experiment, f is the frequency of the waveform (44 Hz), and t is the time. The voltage waveforms of experiment and simulation are shown as follows.

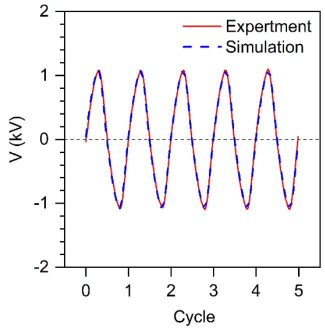

Supplementary Figure 5 | Voltage waveforms of experiment and Fourier series approximation input in DBD microplasma simulation. Experimental voltage waveform (red solid line) is the same as Fig. 2d. Simulation voltage waveform (blue dash line) is used in the COMSOL model and the equivalent circuit model as the power supply.

Hence the expression of the voltage waveform is used as the input of the two models as follows. The simulation

setup of COMSOL model and equivalent circuit model are explained, respectively.

COMSOL model

A one dimensional, axially-symmetric geometry is adopted in consideration of the configuration of the DBD microplasma device, which is described in the method section of the manuscript. A plasma model is selected as the only physics model. The reactions of argon considered in simulation are list as follows.

$$e + Ar \rightarrow e + Ar$$

$$e + Ar \rightarrow e + Ar^*$$

$$e + Ar^* \rightarrow e + Ar$$

$$e + Ar \rightarrow 2e + Ar^+$$

$$e + Ar^* \rightarrow 2e + Ar^+$$

$$Ar^* + Ar^* \rightarrow e + Ar + Ar^+$$

$$Ar^* + Ar \rightarrow Ar + Ar$$

The control equations are provide by the built-in plasma model of COMSOL, as same as the references.[4] The glass capillary is set to the zone of charge conservation. The boundary conditions are that the tungsten is set as the metal contact and the wall that is the voltage waveform input as well, the inner of capillary is set as the wall and the surface charge accumulation, and the copper electrode outside of capillary is treated as the ground. The initial values are set to electron density $n_{e0}=10^6$ [1/m^3], mean electron energy $\varepsilon_0=5$ [V], electric potential $V=0$ [V].

Equivalent circuit model

The discrete components in the equivalent circuit are of the parameters in Supplementary Table 2 as follows.

Name	Parameter value	Name	Parameter value
C_d	5.17 pF	R_p	0.8 GΩ
C_{g1}	0.8 pF	R_v	1 GΩ
C_{g2} (capacitance)	3.5 pF	R_0 (capacitance)	58 pF
C_{g2} (resistance)	1 GΩ	R_0 (resistance)	50 Ω

Supplementary Table 2 | The parameters of the discrete components. The values of capacitance and resistance in the equivalent circuit model of microplasma.

Where C_d is the capacitance of the dielectric barrier (glass), C_{g1} and C_{g2} are the capacitance of the gap between the tungsten wire and the capillary. The change of the capacitance in the process of the discharge can be simulated, technically, by means of disconnecting and connecting two capacitors, C_{g1} and C_{g2}. Switch1 is normally closed and switch2 is normally open. Hence C_g is equal to C_{g1} plus C_{g2}. The capacitance of DBD microplasma device could be measured by LCR meter as around 2.35 pF, in which the C_d is calculated as 5.17 pF via Supplementary Equation 8.

$$C_d = \frac{2\pi\varepsilon_0\varepsilon_r l}{\ln\left(\left(R+x\right)/R\right)} \tag{8}$$

Where $\varepsilon_0=8.854\times10^{-12}$ Fm^{-1} is the permittivity of vacuum, $\varepsilon_r=4.3$ is the relative permittivity of the glass, $R=0.485$

mm is the inner radius of the capillary, x=0.285 mm is the thickness of the glass, l=10 mm is the width of the copper electrode outside of the capillary. The optimal parameters of C_{g1}, C_{g2}, and R_p were selected by running the simulation until the consistency between the experimental results and the simulation was achieved. R_0 is the inner impedance of the TENG measured by LCR meter. The waveform of the voltage as power supply in the circuit complies with Supplementary Equation 7.

Supplementary Note 5: Electric characteristic measurement and analysis of other types of discharges

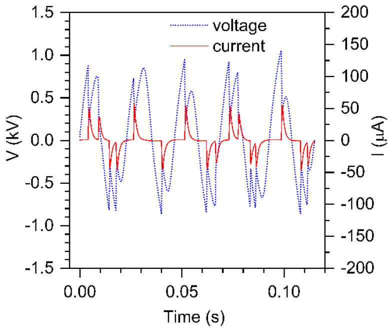

Supplementary Figure 6 | Voltage and current waveforms of a typical discharge using the device in the inset of Fig. 4i (type 3). The device consists of a stainless steel capillary tube as a microhollow electrode, and the copper foil as the other electrode. The gap between them is set as 0.1mm.

The configuration of the stainless-steel capillary tube is that the outer diameter 0.30mm, the inner diameter is around 0.13mm, and the length is 13mm. And there is a gap (~100s μm) between the end of the capillary tube and the copper electrode. The picture of this device is shown in the inset of Fig. 4l. Though the configuration of this device is very similar to the MHCD in the literature[5], we still consider this discharge as a type of microspark because of no MHCD behavior observed. One or more discharges occur per cycle of the voltage, when the TENG directly driving the device. For one cycle, in the beginning there is no discharge when the voltage rises, and then at the voltage of breakdown the discharge occurs that means one or two immediate hops of the current meanwhile voltage drops down due to the charge transfer. These waveforms are more similar to those microspark discharge exhibited in Fig. 2g.

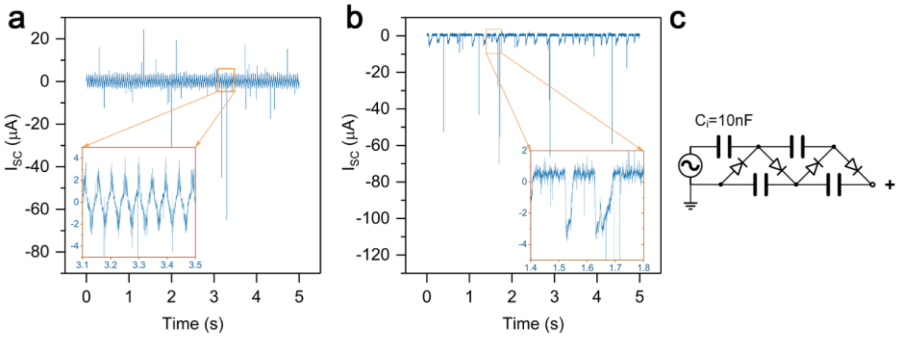

Supplementary Figure 7 | Current waveform of dielectric barrier discharge (DBD) in air. (a) Directly driven by triboelectric nanogenerator (TENG). **(b)** Driven by a voltage multiplier circuit **(c)**.

The device consists of a capillary (Ø$_{outer}$ 0.88 mm, Ø$_{inner}$ 0.4 mm), a copper wire (Ø0.05 mm) as an electrode and a band of copper foil with width of 10 mm wrapped around the capillary. The air gas flow rate is 0.4 liter per minute (l/min). The rotation speed of the FR-TENG is set to 200 rpm. The capacitance of each capacitor in the voltage multiplier circuit is 10 nF.

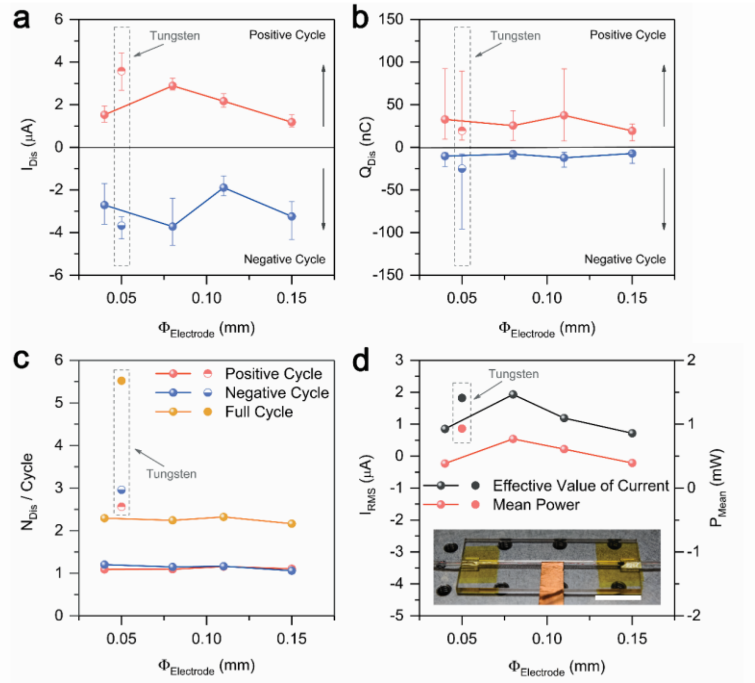

Supplementary Figure 8 | Discharge characteristics of microplasma with different wire electrodes. Statistical characteristics change with the diameter of copper wire electrodes from 0.04mm to 0.15mm, in addition of a tungsten wire electrode with the diameter of 0.05mm. (**a**, **b**) Peak of discharge current and charge in positive and negative cycle discharge, where sphere indicates median, and error bar means the range from 10% to 90% of the whole sampling data. (**c**) Time averaged number of discharge per cycle. (**d**) Time averaged effective value of current and mean power (scale bar, 20 mm).

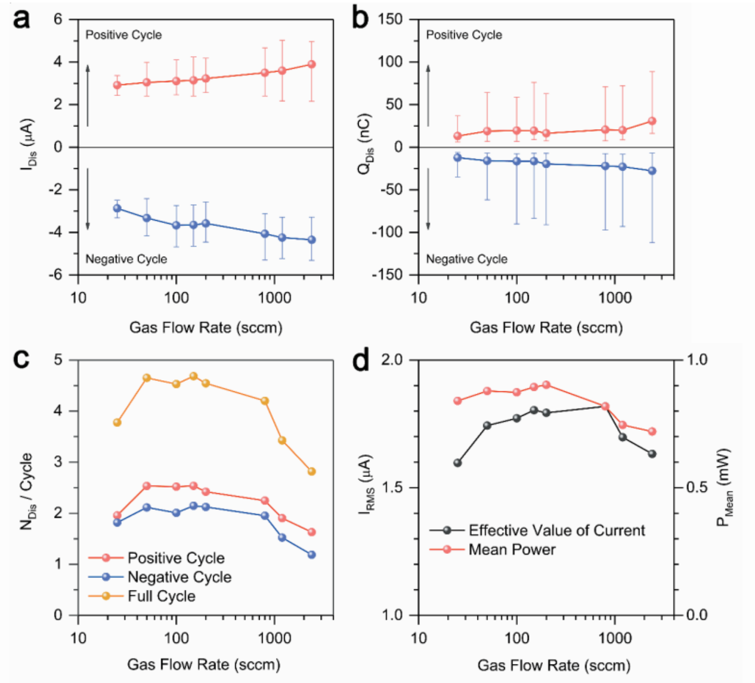

Supplementary Figure 9 | Discharge characteristics of microplasma with different gas flow rate. Statistical characteristics change with gas flow rate of argon from 25 to 2400 sccm, adopting the tungsten wire electrode in the capillary with outer diameter of 1.54mm. There is nearly no discharge in

atmospheric air without argon. (**a, b**) Peak of discharge current and charge in positive and negative cycle discharge, where sphere indicates median, and error bar means the range from 10% to 90% of the whole sampling data. (**c**) Time averaged number of discharge per cycle. (**d**) Time averaged effective value of current and mean power.

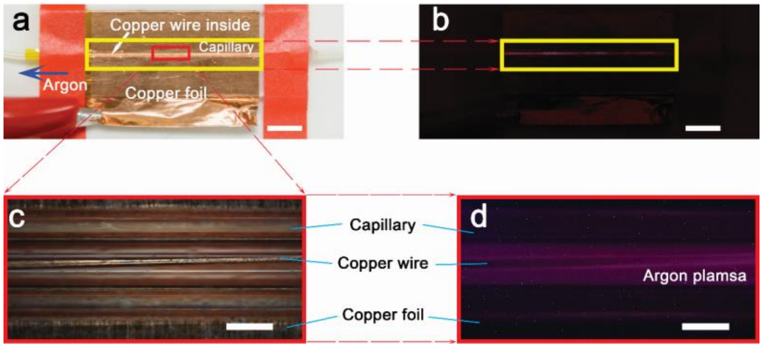

Supplementary Figure 10 | Photograph of dielectric barrier discharge (DBD) capillary microplasma device and luminescence. A glass capillary (Ø0.88 mm) with a copper wire (Ø0.04 mm) inside lays on copper foil, and argon flow through capillary. (**a**) Device photograph in light room (scale bar, 10 mm). (**b**) Corresponding to image (a), luminescence of argon microplasma in dark room (Nikon D700 @ 70mm, ISO 800, 13sec, *f*/2.8) (scale bar, 10 mm). (**c**) Zoom-in photograph of capillary where red rectangle in image (a), was taken with a microscopy (Zeiss Axio) (scale bar, 500 μm). (**d**) Corresponding to image (c), argon microplasma photograph in microscopy (scale bar, 500 μm).

Supplementary References

1. Meng, F., Li, X. & Duan, Y. Chip-based ingroove microplasma with orthogonal signal collection: new approach for carbon-containing species detection through open air reaction for performance enhancement. *Sci. Rep.* **4**, 4803 (2014).
2. Weagant, S., Chen, V. & Karanassios, V. Battery-operated, argon-hydrogen microplasma on hybrid, postage stamp-sized plastic-quartz chips for elemental analysis of liquid microsamples using a portable optical emission spectrometer. *Anal. Bioanal. Chem.* **401**, 2865-2880 (2011).
3. Eijkel, J. C. T., Stoeri, H. & Manz, A. A dc microplasma on a chip employed as an optical emission detector for gas chromatography. *Anal. Chem.* **72**, 2547-2552 (2000).
4. Wang, H., Li, H. & Wen, L. One dimensional simulation of microscale DBD plasma. In: *10th IEEE International Conference on Nano/Micro Engineered and Molecular Systems (IEEE-NEMS 2015)*, 515-520 (2015).
5. Sankaran, R. M. & Giapis, K. P. Hollow cathode sustained plasma microjets: characterization and application to diamond deposition. *J. Appl. Phys.* **92**, 2406-2411 (2002).
