# Supplementary material for: Triboelectric microplasma powered by mechanical stimuli
Source: Nat Commun. 2018 Sep 13;9:3733. doi: 10.1038/s41467-018-06198-x (PMC6137053; doi:10.1038/s41467-018-06198-x)
Supplement: Supplementary file 2 — Description of Additional Supplementary Files [file 41467_2018_6198_MOESM2_ESM.pdf]

Description of Additional Supplementary Files

File Name: Supplementary Movie 1
Description: Microplasma luminescence of patterned electrode directly driven by FR-TENGs (freestanding rotary triboelectric nanogenerators)

File Name: Supplementary Movie 2
Description: DBD (dielectric barrier discharge) plasma simulation

File Name: Supplementary Movie 3
Description: Microspark discharge directly driven by FR-TENG

File Name: Supplementary Movie 4
Description: Change of discharge mode and status from microspark to DBD plasma

File Name: Supplementary Movie 5
Description: A plasma disk driven by FR-TENGs
